# Rapid modulation in music supports attention in listeners with attentional difficulties

**DOI:** 10.1038/s42003-024-07026-3

**Published:** 2024-10-23

**Authors:** Kevin J. P. Woods, Gonçalo Sampaio, Tedra James, Emily Przysinda, Bernardo Cordovez, Adam Hewett, Andrea E. Spencer, Benjamin Morillon, Psyche Loui

**Affiliations:** 1 Brain.fm, Brooklyn, NY USA; 2 Wesleyan University, Middletown, CT USA; 3 RootBio LLC, Pacifica, CA USA; 4 Boston University Chobanian & Avedisian School of Medicine, Boston, MA USA; 5 Aix Marseille Université Inserm, INS, Institut de Neurosciences des Systèmes, Marseille, France; 6 Department of Music, College of Arts, Media, and Design, Northeastern University, Boston, MA USA

**Keywords:** Attention, Cognitive control, Human behaviour

## Abstract

Background music is widely used to sustain attention, but little is known about what musical properties aid attention. This may be due to inter-individual variability in neural responses to music. Here we find that music with amplitude modulations added at specific rates can sustain attention differentially for those with varying levels of attentional difficulty. We first tested the hypothesis that music with strong amplitude modulation would improve sustained attention, and found it did so when it occurred early in the experiment. Rapid modulations in music elicited greater activity in attentional networks in fMRI, as well as greater stimulus-brain coupling in EEG. Finally, to test the idea that specific modulation properties would differentially affect listeners based on their level of attentional difficulty, we parametrically manipulated the depth and rate of amplitude modulations inserted in otherwise-identical music, and found that beta-range modulations helped more than other modulation ranges for participants with more ADHD symptoms. Results suggest the possibility of an oscillation-based neural mechanism for targeted music to support improved cognitive performance.

## Abstract (web summary)

Rapid temporal modulation added to music affects attentional performance and brain activity; parametric manipulation of this added modulation reveals rates that differentially affect individuals with above-average attentional challenges.

## Introduction

Music often has practical uses beyond esthetic appeal^1^, and from mothers’ lullabies to laborers’ work songs, the music we make to fill these roles reflects its function^2,3^. One possible use of music is to aid cognitive performance^4–6^. This has become increasingly important with the shift to knowledge-work^7–10^, along with widespread adoption of technologies like streaming and personal audio. To date, many different kinds of music have been used to aid focus in the workplace^11^. The diversity in music used for focus may reflect individual differences in cognitive styles: for example, personality differences are associated with the ability to sustain attention^12–14^. Preference and familiarity also contribute to effects of music on cognition^15–17^.

Another factor deserving special consideration is an individual’s ability to focus. Prior work has shown that auditory stimulation can aid performance in individuals with ADHD^18–21^. This has been explained by optimal stimulation theory, which poses that some individuals, specifically those with ADHD, require more stimulation than others to function best^22–25^. However, all these cases compare stimulation (music or noise) to silence, and no studies of this kind to date have used experimental conditions with different types of music.

To measure sustained attention, we used the Sustained Attention to Response Task (SART), a computerized task in which participants respond via keypress to a sequentially-presented series of numerical digits, but withhold responses to infrequent (10%) numerical targets. Performance on the SART has shown sensitivity and ecological validity in measuring sustained attention ^26,27^.

**Fig. 1:**
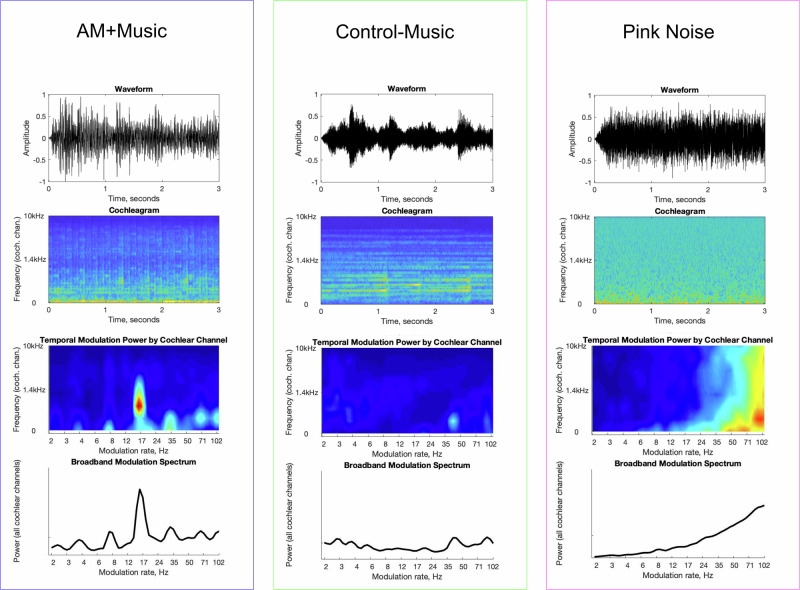
Acoustic analyses of auditory stimuli used in Experiments 1–3. Analysis of a 30-s excerpt from each stimulus type used in Experiments 1–3. Pressure over time (top row) first undergoes frequency decomposition via cochlear filtering. The energy in each cochlear channel varies over time (depicted on the cochleagram, 2nd row). These envelope fluctuations are then frequency-decomposed to produce a modulation spectrum representation (3rd row). The broadband modulation spectrum (bottom row) is the sum of modulation spectra across the cochlear channels. This broadband modulation shows peak in the AM + Music condition only, where rapid modulation was added to the music.

We first compared SART performance (Experiment 1) under three types of background acoustic conditions: AM + Music (i.e., music with fast amplitude modulations), Control − Music (with slow amplitude modulations), and Pink Noise. The AM + Music had fast modulations added that do not usually occur in music, and acoustic analyses (Fig. 1) showed that despite similar frequency content, the tracks differed in the modulation domain due to this added modulation. We then used the same stimuli and task in experiments with fMRI (Experiment 2) and EEG (Experiment 3). Finally, additional behavioral experiments (Experiments 4A and 4B) tested the effects of modulation on sustained attention in an acoustically controlled manner, on groups of participants split by their level of attentional difficulty.

## Results

### Experiment 1: Sustained Attention to Response Task (SART)

**Fig. 2:**
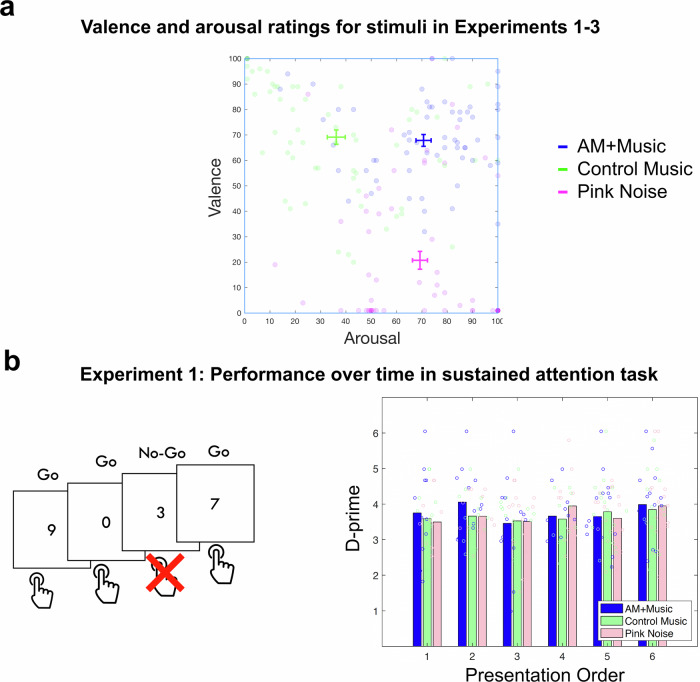
Stimulus valence and arousal ratings and performance over time in the Sustained Attention to Response Task (SART). **a** Valence and Arousal ratings for the music used in Experiments 1-3. *N* = 62. **b** Performance on the SART in a separate group of participants (*N* = 83) by music condition over time (as indicated by trial block). Left panel shows a schematic of SART trials, which were performed under 3 music conditions in Experiment 1. Each participant completed 6 blocks (2 of each music condition) presented in random order.

Participants were recruited and tested online via Amazon’s Mechanical Turk web service. In Experiment 1A, 62 participants rated the AM + Music, Control − Music, and Pink Noise for valence and arousal. Figure 2a shows means and standard errors of valence and arousal ratings from Experiment 1A. In Experiment 1B, another 83 participants completed the SART under the three acoustic conditions. Figure 2b shows performance on the SART for each condition depending on the order it was presented in the experiment.

Valence and arousal ratings for all three sound stimuli were entered into a multivariate one-factor MANOVA, with the within-subjects factor of Music (3 levels: AM + Music, Control − Music, and Pink Noise). The MANOVA indicated a significant large difference in valence and arousal between the different groups, *F*(4, 364) = 56.63, *p* < 0.001, Wilk’s lambda = 0.38, partial *η*^2^ = 0.38. Participants rated AM + Music as positive in valence and high in arousal, Control − Music as positive in valence and low in arousal, and Pink Noise as low in valence but high in arousal.

Since the SART is a test of sustained attention over time, performance on the SART over time was analyzed with a repeated measures mixed-effects ANOVA with the dependent variable of d-prime. The independent variables were again the within-subjects factor of Music (3 levels: AM + Music, Control − Music, and Pink Noise) and trial block (blocks 1 to 6, with 200 trials per block, see Methods). The order of presentation of the musical stimuli, which was counterbalanced in this within-subjects design study, was modeled as a between subjects variable. As participants were allowed to adjust the volume of their own headphones as they were tested online, the chosen volume (determined using custom online volume testing method described in Supplementary Materials: Listener Volume Settings) was also used as a covariate. Results showed a significant Music by trial-block interaction (*F*(38,38) = 2.024, *p* = 0.016, *η*_p_^2^ = 0.669). There was also a significant Music by presentation-order interaction (*F*(10, 150) = 2.298, *p* = 0.015, *η*_p_^2^ = 0.133). Follow-up visualizations of the Music by trial-block and presentation-order effects on d-prime showed that the effects were driven by primacy of the AM + Music: participants who received the AM + Music as the first music condition (Presentation orders 1 and 2 in Fig. 2b) significantly outperformed those who received the other music conditions first.

The direction of this interaction—with fast modulations benefiting the first block of SART performance in particular—aligns with our hypothesis that arousal in music can affect sustained attention, with effects being observed almost immediately upon first experiencing AM in music. To better understand the neural bases of this effect, we ran the same task and background conditions in neuroimaging experiments, with fMRI (Experiment 2) and EEG (Experiment 3), looking not only at the brain’s response to the different types of music, but also task-related activity.

### Experiment 2: SART fMRI during background music

In an fMRI study, 34 participants completed the SART under the same three background music conditions used in Experiment 1: AM + Music, Control − Music, and Pink Noise.

**Fig. 3:**
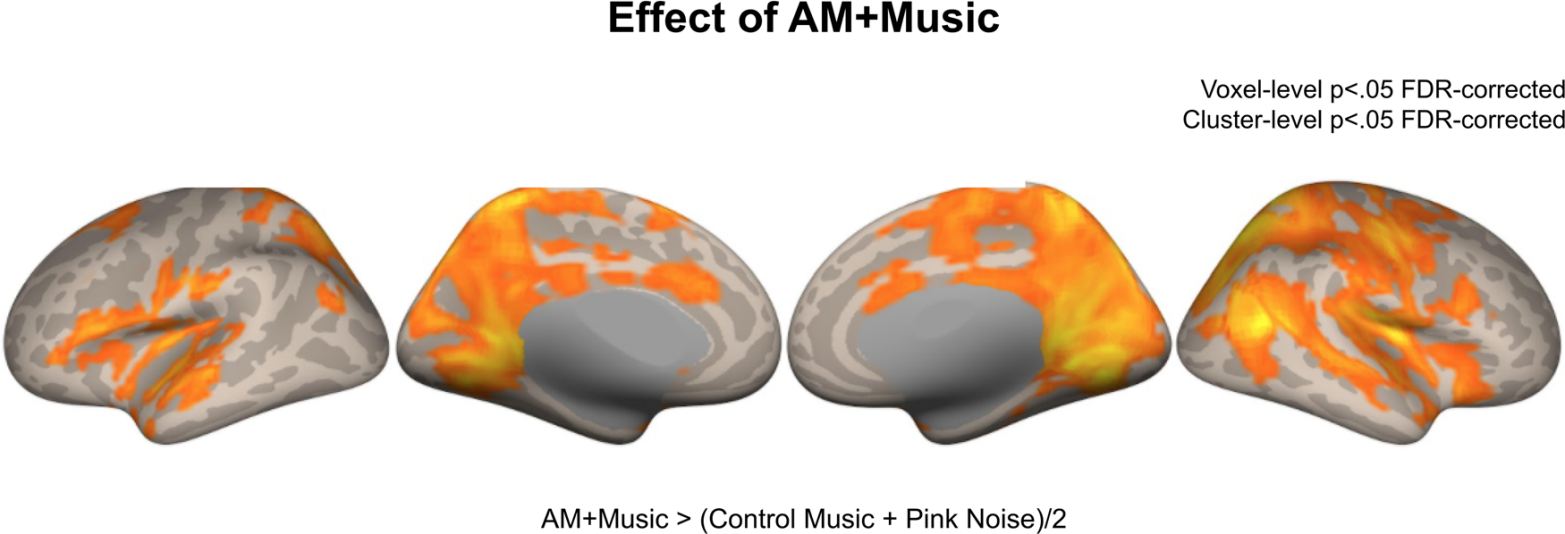
fMRI results comparing AM + Music, Control − Music, and Pink noise during the Sustained Attention to Response Task (SART). Contrast between AM + Music and the average of Control − Music and Pink Noise while participants performed a sustained attention (SART) task in Experiment 2. Higher levels of activity for AM + Music are widespread across many regions.

A within-subjects ANOVA comparing overall brain activity during the three conditions showed significantly higher activation during the AM + Music condition than in the other two conditions (*p* < 0.05 FDR-corrected) in multiple regions including the bilateral superior temporal lobes, frontal lobes, parietal lobes, and mesial and lateral occipital cortices, encompassing the default mode, executive function, and salience networks (Fig. 3). Additional areas that survived cluster-wise FDR correction included the bilateral middle frontal gyri and frontal operculum, medial prefrontal cortex, bilateral temporal lobes, and lateral occipital cortex. The latter regions are part of the default network and the ventral attention network^28^, and their additional involvement is consistent with the role of the salience network in facilitating attention resources and accessing the motor system upon the detection of salient events. No other contrast showed positive suprathreshold clusters (all *p* > 0.05 FDR-corrected).

### Higher activity in motor network linked to successful behavior during AM + Music

**Fig. 4:**
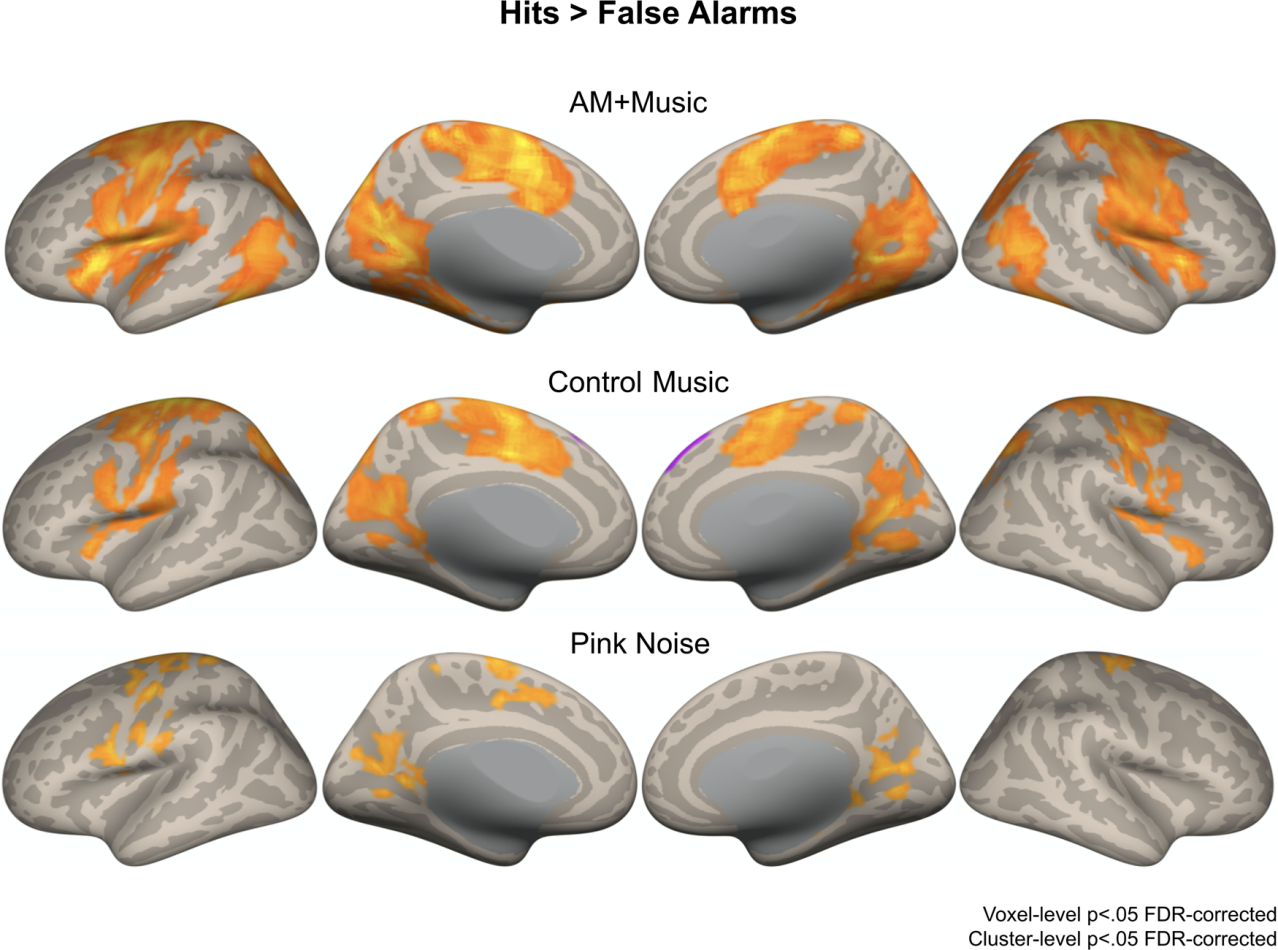
fMRI results comparing hits and false alarm trials from the Sustained Attention to Response Task (SART) during AM + Music, Control − Music, and Pink Noise. Brain activity during Hits contrasted against False Alarms on the SART showing increased activity during correct trials despite similar motor output, centering around the sensorimotor network and the salience network during AM + Music compared to Control − Music and Pink Noise. Warm colors show greater activity for Hits; cool colors show greater activity for False Alarms.

To relate behavior to brain activity during the different music tracks, we fit separate parametric models in Statistical Parametric Mapping 12 (SPM12)^29^ for hit trials and false alarm trials for each auditory condition. Brain activity during successful responses, quantified as a contrast between activity during hits and activity during false alarms, showed significantly higher activity during the hits in all auditory conditions (Fig. 4). Importantly, the Hits vs. FA contrast at the *p* < 0.05 FDR-corrected level showed more significant clusters during AM + Music than during any other condition (Control − Music, Pink Noise). These clusters centered around the sensorimotor network (supplementary motor area, precentral gyrus, the salience network (anterior cingulate cortex, anterior insula), and the visual association network (lateral and mesial occipital regions). These differences were observed despite overall similar hit and FA rates across the auditory conditions (one-way ANOVAs all Fs < 1). While the interaction between auditory conditions and groups was not significant at the whole-brain level, participants showed more activity for AM + Music than for the other auditory conditions during Hit trials compared to FA trials. Together, these results show that AM + Music is linked to higher levels of brain activity in multiple networks, especially during successful behavioral performance of a sustained attention task. These individual differences in brain activity could underlie music-induced differences in performance from Experiment 1.

Since the musical stimuli were highly rhythmic, we expected that they might affect rhythmic activity in the brain, and that music-induced changes in such activity could provide further insight into the mechanisms by which music affects sustained attention. To capture rhythmic neural activity and relate it to stimulus rhythms with high temporal precision, we turned to an EEG study with the same stimulus and task conditions as the fMRI study reported above.

### Experiment 3: SART EEG during background music

Forty participants had their EEG recorded while they performed the SART task under the same three background music conditions used in Experiments 1 and 2: AM + Music (containing fast modulation rates; more arousing), Control − Music (containing slow modulation rates; less arousing), and Pink Noise. Hit and FA rates were overall similar across the auditory conditions (one-way ANOVAs all Fs < 1).

### Stimulus-brain coupling EEG shows phase locking at peak frequencies of amplitude modulation

**Fig. 5:**
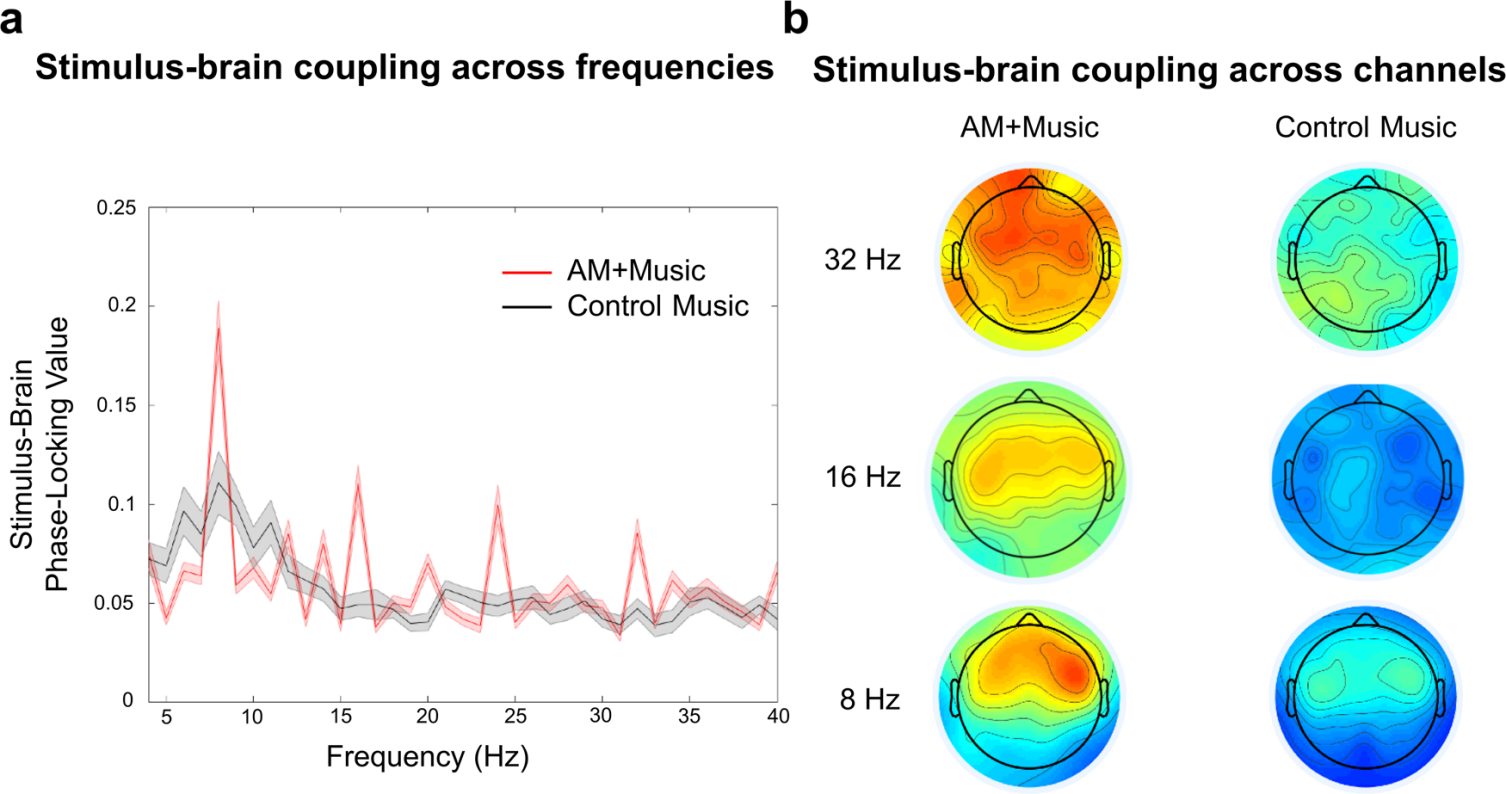
EEG reveals stimulus-brain coupling. **a** For Experiment 3, we conducted hertz-by-hertz Morlet wavelet analysis of each acoustic stimulus (amplitude-modulated and control) and evaluated the phase-locking value (stimulus-brain coupling) between acoustics of the stimulus and the EEG. Red trace = Mean ± SE Phase Locking Values (PLVs) of frontocentral electrodes for AM + Music. Black trace = Mean ± SE PLVs of the same electrodes for the Control − Music. Results show peaks of phase-locking activity at the note rates and its harmonics (8, 16, 24, 32 Hz) as well as the amplitude-modulated frequencies (12, 14, 16 Hz). **b** Topographic distributions of the peaks of phase-locked activity at 8, 16, and 32 Hz for amplitude-modulated and control stimuli.

We assessed coupling between the EEG and the acoustic signal by computing the stimulus-brain phase-locking value (PLV) for every frequency (in 1-Hz bins). During the AM + Music, stimulus-brain PLV showed prominent peaks at 8, 12, 14, 16, 24, and 32 Hz (Fig. 5a). Since the AM + Music was at 120 bpm (i.e., quarter-notes at 2 Hz), 8, 16, 24, and 32 Hz are harmonics of the note rate, previously observed to entrain cortical activity^30^, while 12, 14, and 16 Hz reflect the amplitude-modulated frequencies.

PLV at 8 Hz during AM + Music was strongest at frontal recording sites. In contrast, PLV during Control − Music was much lower and less sharply tuned, reflecting less focused neural tracking of acoustic rhythms when listening to Control − Music. Looking across the whole brain (Fig. 5b), PLV at 8 Hz was stronger in the AM + Music condition, even over the same frontal recording sites. We computed an effect size (Cohen’s *d*) measure for every 4-Hz bin (thus capturing 8, 12, 16, 20, 24, 28, and 32 Hz) across the full frequency spectrum, comparing AM + Music against Control − Music, averaging across all frontal, central, and parietal electrodes. This resulted in a Cohen’s *d* of 3.74, confirming a highly statistically significant difference between AM + Music and Control − Music at these frequencies of interest. In contrast, the same analysis for 1-Hz bins showed a Cohen’s d of 0.227, suggesting higher stimulus-brain coupling at multiples of 4-Hz, consistent with the note rate and with the amplitude-modulation rates in the music.

### Phase-locking over time

**Fig. 6:**
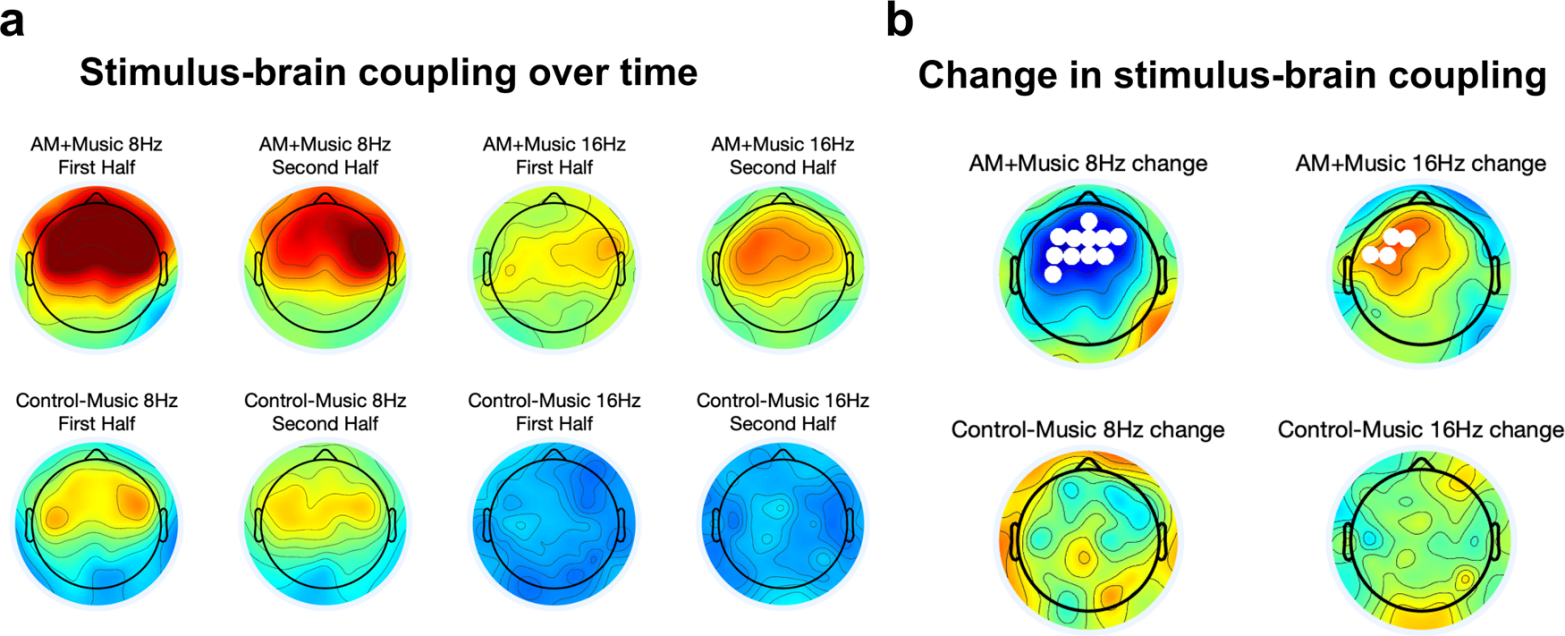
Stimulus-brain coupling over time for AM + Music and Control − Music. **a** Stimulus-brain coupling over time for AM + Music and Control − Music at 8 Hz and at 16 Hz, showing higher PLV for AM + Music than for Control − Music. **b** Change in stimulus-brain coupling over time for AM + Music and Control − Music at 8 Hz and 16 Hz. White dots denote statistically significant channels (*t*-test comparing late trials vs. early trials, FDR-corrected).

We compared stimulus-brain PLV for AM + Music and Control − Music at 8 Hz and at 16 Hz, showing higher PLV for AM + Music than for Control − Music. If AM + Music was responsible for improvement in attention over time by phase-locking to brain activity that was important for behavior, then there should be an increase in PLVs between AM + Music and EEG over time, which would be detectable by comparing late trials (i.e., in the second half of the EEG experiment) against early trials (in the first half). We broke down the PLV by the first and second halves of the EEG recording session, and observed that over the duration of the experiment there was an increase in PLV for AM + Music in the beta band, specifically at 16 Hz (Fig. 6a), that was not observed for Control − Music. This increase in phase-locking over time was statistically significant over left frontal channels (*t*-tests comparing late trials vs. early trials, significant channels at the *p* < 0.05 FDR-corrected level shown in Fig. 6b). In the theta band, specifically at 8 Hz, there was a decrease in PLV over time for AM + Music that was not observed for Control − Music (Fig. 6a, significant channels from *p* < 0.05 FDR-corrected *t*-test comparing late trials vs. early trials, Fig. 6b).

If people who have difficulty focusing have distinct needs for focus-music, there is an opportunity to develop a targeted solution for those who could use it most. We were thus interested to see if music with different acoustic properties would affect people differently depending on their attentional capacity. If so, people with attentional deficits, such as symptoms of ADHD, may need specifically designed focus music. We hypothesize that amplitude modulation in music can affect performance differently in people with different levels of attentional difficulties^19,21,31^. Specifically, we hypothesize that beta-range modulation should benefit sustained-attention performance over time with music modulated at rates from 12–20 Hz.

### Experiment 4: parametric manipulation of modulation rate and depth

**Fig. 7:**
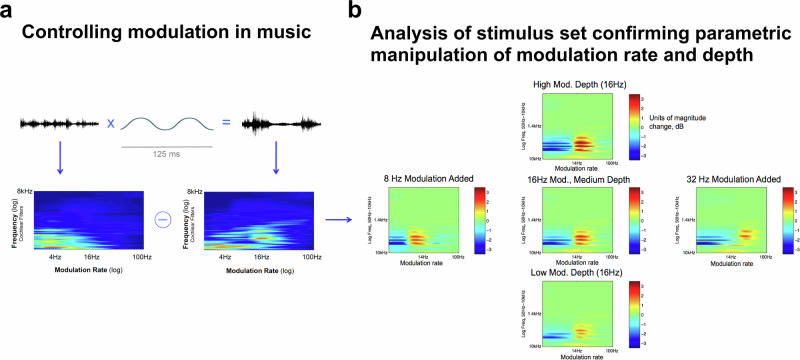
Controlling modulation in music stimuli. Modulation in sound can be summarized by the modulation spectrum: The vertical axis on these 2D plots depicts audio frequency (0–8 kHz). The horizontal axis shows the amplitude modulation spectrum (amplitude fluctuations from 0 to 100 Hz). **a** Applying modulation to music results in a modulation spectrum with a peak corresponding to the rate of the added modulation. Upper: The pressure wave is multiplied by a modulator (in this case 16 Hz) to produce a modulated signal. Lower: This pressure wave is bandpass filtered, and fluctuation rates in each channel are shown in the modulation spectrum. **b** Validation of stimulus manipulations used in Experiment 4. The stimulus space is illustrated by these panels, each of which shows the difference in modulation spectrum (normalized power) between an unmodulated track and the experimental conditions derived from this track. The rate of added modulation increases moving rightward, while depth increases moving upward. The absence of differences elsewhere on the modulation spectrum shows that the experimental conditions were altered in a controlled way, with modulation properties different from the original only as specified (stimulus validation).

The stimuli in Experiments 1–3 were taken from commercially available focus music that one might encounter if searching for music to work to. They were chosen to have a dramatic difference in modulation characteristics, as was apparent in acoustic analyses (Fig. 1). However, they also differed in low-level acoustic properties (e.g., overall spectral balance) as well as musical features (e.g., tonality, instrumentation). To control for these differences and to isolate the effect of amplitude modulation, we developed new stimuli in which otherwise identical music was manipulated to impose modulation of varying rates and depths (Fig. 7).

Since Experiments 2 and 3 revealed effects on brain activity associated with attention and attentional challenges such as ADHD^27^, we additionally sought in this final experiment to split our participants according to their level of attentional difficulty (via the Adult ADHD Self-Report Scale, or ASRS). We expected that high-ASRS (i.e., more ADHD-like) participants would benefit more from music that had added amplitude modulation specifically in the beta range (12–20 Hz), since this oscillatory regime is associated with sustained attention.

### Parametrically testing the effects of amplitude modulations: rate and depth

Acoustic amplitude modulations are known to drive neural oscillations, i.e., to induce a selective amplification of neural activity at this frequency. This effect occurs along the auditory pathway but also in cortical networks such as the attentional network^32–35^, and may thereby impact cognitive processes. We chose to test rates of 8, 16, and 32 Hz for two reasons: First, these fall within ranges of distinct neural oscillatory regimes that are known to have different functions in the brain. Alpha (8–12 Hz), beta (14–25 Hz) and gamma (25–100 Hz) rhythms are three such ranges, and our experimental conditions using 8, 16, and 32 Hz modulation thus fall into each of these oscillatory regimes. Second, these rates were chosen to correspond to note values. As we used music that was composed at 120 beats per minute (2 Hz), amplitude modulation rates at 8, 16, and 32 Hz correspond to 16th, 32nd, and 64th notes respectively.

Beta-band cortical activity is implicated in the maintenance of sensorimotor or cognitive states^36^ and top-down processing in general^37^ including attentional control^38,39^. Moreover, in spatial attention tasks, beta-band increase is observed in the hemisphere that represents the attended stimulus, resulting in enhanced processing of the attended stimulus^40^. In contrast to the other bands, stimulating neural activity in the beta band with the 16 Hz modulation condition thus seemed most likely to confer a performance benefit on sustained attention.

Depth of modulation refers to how heavily the sound is modulated, rather than the rate of modulation. A maximal depth of modulation would mean that sound energy is reduced to zero at the troughs of the applied modulating waveform, while a very low depth would mean barely-perceptible modulation. While a greater modulation depth is expected to impact neural oscillations more strongly, beyond a point the underlying music suffers esthetically as the sound becomes distracting due to increased auditory salience from the sudden changes in loudness over time^41^.

One hundred seventy five participants completed two SART experiments online (4A and 4B) each with four conditions, testing modulation depth and modulation rate separately. In both cases two groups of participants each heard four conditions, presented for 5 min each in counterbalanced order. The first group of participants heard no-modulation, 8 Hz medium-depth, 16 Hz medium-depth, and 32 Hz medium-depth modulation. The second group heard no-modulation, 16 Hz low-depth, 16 Hz medium-depth, and 16 Hz high-depth modulation. Thus, rate and depth (4 conditions each) were tested on separate groups of participants, but each participant heard all four possible rates or all four possible depths for five minutes each. This limited the duration of each condition, but maximized statistical power in comparing across all the conditions in one dimension of the modulation spectrum, while controlling for intrinsic between-subject differences in performance that are unrelated to our conditions of interest.

Valence and arousal ratings for all six sound stimuli were entered into a multivariate ANOVA (as in Experiment 1), with the within subjects factor of Music (6 levels: No Modulation, 16 Hz Modulation at Low, Medium, and High Depth, 8 Hz and 32 Hz at Medium Depth). A main effect of Music was observed on both valence and arousal (valence: *F*(5305) = 32.7, *p* < 0.001; arousal: *F*(5305) = 7.1, *p* < 0.001).

**Fig. 8:**
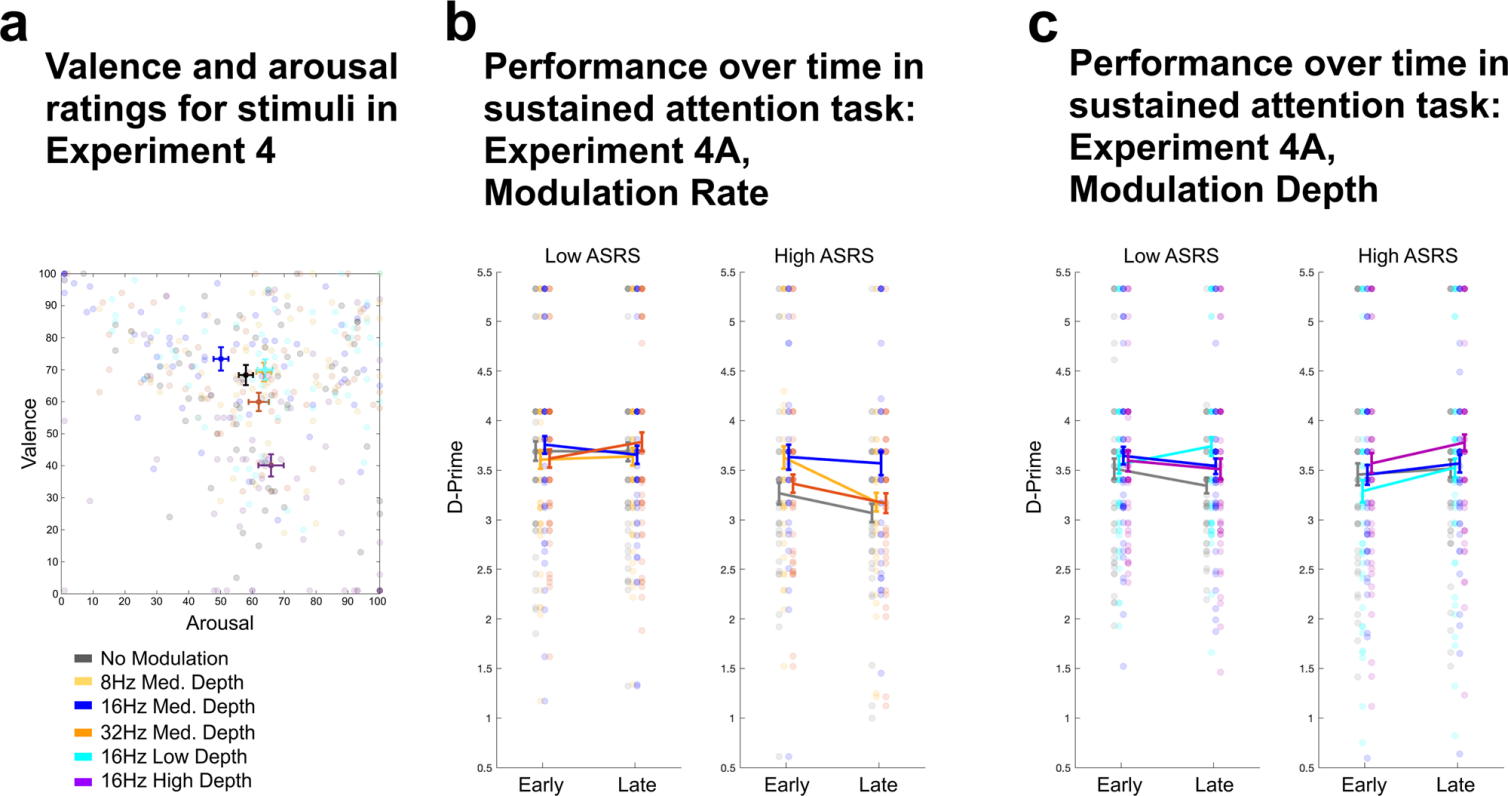
Stimulus valence and arousal ratings and performance over time in the Sustained Attention to Response Task (SART) for stimuli parametrically manipulated in modulation rate and depth. **a** Valence and Arousal ratings for the music used in Experiment 4. *N* = 62 overall (same participants as Experiment 1). Error bars represent ±1 within-subject SEM. **b** Performance on the SART for varying rates of added modulation. *N* = 82 overall, *N* = 46 and *N* = 36 in the Low and High ASRS groups respectively. **c** Performance on the SART for varying depths of added modulation. *N* = 93 overall, *N* = 48 and *N* = 45 in the Low and High ASRS groups respectively. In Experiments 4A and 4B, each participant completed 4 blocks (the music conditions) presented in random order; the blocks are overlaid in the figures. Error bars represent ±1 within-subject SEM, and thus do not reflect the variability in overall performance across participants.

Overall performance (d-prime) for the SART in the rate and modulation depth experiments are visualized in Fig. 8b, c separately for high- and low-ASRS groups, but since ASRS is a continuous measure of individual differences, we treated ASRS score as a continuous covariate in a repeated measures mixed-effects ANOVA with the dependent variable of d-prime difference relative to the no-modulation control condition. Independent variables were again the within-subjects factor of modulation (3 levels for each within-subjects manipulation: 8 Hz, 16 Hz, and 32 Hz for the modulation-rate manipulation; low, medium, and high depth for the modulation-depth manipulation) and trial block (early and late blocks respectively).

The modulation rate experiment (Fig. 8b) showed a significant three-way interaction (*F*(1,80) = 4.03, *p* = 0.048, *η*_p_^2^ = 0.048) between modulation rate (quadratic effect, showing highest performance in the medium-rate 16 Hz modulation), block (linear effect, showing higher performance in the early block), and ASRS score (higher performance in low-ASRS participants). Participants who scored higher on the ASRS performed significantly better over time with the 16 Hz modulation than other participants in other modulation conditions. No significant main effects or interactions were observed for the modulation depth experiment.

## Discussion

Performance on a variety of everyday tasks requires sustained attention. Cognitive failures, specifically failures in sustained attention, are linked to mind wandering, which is associated with decreased productivity and happiness^42^. Music is widely used to help with sustained attention, but a variety of music is used with little agreement on what works best. Here, we show that a specific type of amplitude-modulated music (AM + Music) was more effective at engaging the salience, executive control, sensorimotor, and visual networks, and at coupling with rhythmic brain activity at multiple frequencies.

Behavioral experiments (Experiment 1) showed that performance on a sustained attention task was affected by background music type, with AM + Music improving performance (albeit only when it occurred early on in the experiment). Participants who received AM + Music as the first music condition (Presentation orders 1 and 2 in Fig. 2b) significantly outperformed those who received the other music conditions first. Fast amplitude modulations presented early on in the experiment benefitted performance such that the first block of SART performance was significantly boosted during AM + Music, aligning with our hypothesis that arousal in music can affect sustained attention, with effects being observed upon first experiencing AM in music.

FMRI and EEG experiments (Experiments 2 & 3) identified some mechanisms behind these effects. The fMRI experiment (Experiment 2) showed higher activity overall in response to AM + Music. AM + Music elicited higher activity in the anterior cingulate cortex and the insula, core nodes of the salience network. Directly contrasting Hits against False Alarms showed a greater extent of correct response-related activity during AM + Music than other conditions, particularly in motor regions. This could indicate that behavioral advantage for AM + Music relates to activity in the motor system, i.e., that the AM + Music could be more effective at priming the motor system to respond correctly when receiving the task-relevant sensory signals from the sensory cortex.

Our EEG experiment (Experiment 3) showed that phase-locking activity was strongest during AM + Music. These results suggested that amplitude modulation could underlie the difference in performance observed in Experiment 1, and the differences in functional network activity observed in Experiment 2. To isolate the effects of amplitude modulation, a final behavioral experiment was conducted using music that differed only in the rate or depth of added modulation (Experiment 4).

Since the functional networks and oscillatory activity revealed in Experiments 2 and 3 respectively bore relevance to the hypothesized neural bases of attentional challenges such as ADHD, this final experiment additionally looked at participants who varied with self-reported ADHD symptoms as captured by the ASRS (attentional difficulty). Experiment 4 found that adding amplitude modulation at 16 Hz resulted in better performance over time that covaried significantly with ASRS score. Thus, adding amplitude modulation to music produced behavioral effects on sustained attention that depended on the listener’s level of attentional difficulty.

We found that the effect of music on sustained attention appears to result from specific interactions between functional differences in brain activity and amplitude modulation patterns in the musical sounds. Individual differences in sensitivity to background music may be attributable to “optimal stimulation level”^43,44^, i.e., the effect of external stimulation on cognitive performance, which differs across individuals, e.g., between introverts and extroverts^12,45,46^ and between groups with and without attentional deficits^25,31,47^. These stimulus-brain interactions could partly explain—in addition to preference and familiarity^15–17^—why people use such different types of music to help them focus, and may suggest routes to more effective personalized focus music in the future.

Experiment 4 revealed that effects of the music varied depending on an individual’s level of attentional deficit, as measured by self-report on the ASRS. While previous studies have observed that individuals with attentional deficits are differently affected by music versus silence^5,14,48^ or noise^12,20,21,49^, here we extend the findings to show that differences between pieces of music are sufficient to affect sustained attention. In our EEG experiment (Experiment 3) the difference between AM + Music and Control − Music suggested that the AM + Music was effective at modifying oscillatory neural activity, particularly in the beta range (12–20 Hz), which has been associated with sustained attention^50^. Similarly in our fMRI experiment 2 (Experiment 2), AM + Music in particular elicited changes in functional networks implicated in attention and ADHD. Thus, in both brain and behavior, rapidly modulated AM + Music showed evidence of affecting sustained attention, aligning with the idea that greater relative levels of beta-band activity are associated with improved performance on sustained attention tasks for individuals with ADHD ^51–53^.

While Experiment 1 suggested that the interaction between music for sustained attention and attentional deficit might depend on arousal, detailed examination of the effects of Fast- and Control − Music on phase-locking activity in the brain suggested that differences in acoustic modulation could underlie performance differences between high-arousal and low-arousal stimuli. In Experiment 4 the stimuli were made to vary only in modulation rate and depth, and the condition with the lowest arousal (16 Hz medium depth condition) produced the best performance in the high-ASRS group. Taken together the experiments suggest that acoustic modulation, rather than arousal per se, is the main driver behind the effects of music on sustained attention. Likewise, valence also cannot explain the differences in task performance. Although the increase in modulation depth from medium to high was sufficient to reverse the valence and arousal ratings of those stimuli, these ratings did not differ substantially between ASRS groups in the conditions that most affected sustained attention.

fMRI results showed higher activity during AM + Music than during other acoustic conditions, with significant effects in widely distributed regions encompassing the salience, executive function, and default mode networks. When contrasting activity between hits and false alarms to isolate successful task-related activity, AM + Music elicited the highest activity in the sensorimotor network, the salience network, and the visual association network. The widespread increases in activity during AM + Music confirm that background music affects sustained attention by influencing multiple interconnected networks that are normally coupled to subserve performance on a variety of cognitive tasks. The default mode and executive function network are typically anticorrelated in activity^54^, with the salience network being a consistent regulator of both other networks^55^. The interplay between salience, executive function, and default mode networks is critical in tasks requiring cognitive control, and this relationship is altered in ADHD by aberrant connectivity between these networks^56^. Here, when listening to AM + Music participants showed increased activity in the three networks. We found particularly strong effects in the mid-cingulate cortex^57^ and the right anterior insula, key components of the salience network, in response to the AM + Music. The simultaneously observed effects in the salience network and the late visual areas / ventral attention network may suggest that the AM + Music affects performance by motivating attention to the visual task. It may also suggest increased functional connectivity among the networks as neural activity becomes coupled to the music. Increased coupling may also explain results from the EEG experiment, which found increased phase-locking to the stimulus at specific frequencies that were targeted by the note rate and the added amplitude modulation patterns of the AM + Music. Within the phase-locking patterns across different frequencies during AM + Music, scalp topography showed the highest phase locking around frontocentral channels at low frequencies (8 Hz) but more widespread activity across the scalp at higher frequencies (32 Hz). These topographical differences in phase-locked activity across different frequencies may be explained by cross-frequency coupling mechanisms which are known to underlie communication across different regions in the brain ^58^.

Our subsequent behavioral results also point to the possibility that the music’s differential impact on oscillatory activity could underlie its effect on sustained attention. In Experiment 4 our stimuli were parameterized by modulation rate and showed a distinct 16 Hz benefit for high-ASRS listeners, which could point to this effect being mediated by oscillatory processes in the brain. Individuals with ADHD have atypical oscillatory activity^35^, including a higher theta/beta ratio^59^. The observed rate-specific effect that covaried with ASRS score in Experiment 4 could indicate that the AM + Music impacts the atypical brain activity in ADHD. Taken together our results suggest that there could be a process by which music at high modulation rates drives brain activity to benefit ADHD-like individuals in particular. Importantly, such modulation rates are not normally found in music, but were added into the AM + Music.

Brain stimulation methods with controlled frequencies have been used to enhance cognitive performance in recent years^60–63^. The literature on rhythmic priming has shown that rhythmic stimulation with sound, specifically using background music, affects the processing of spoken or written language by tuning neural phase entrainment, resulting in superior performance following temporally regular acoustic stimulation^58,64,65^. More generally, sound is another means by which to stimulate the brain^66–68^. Although previous studies on using auditory entrainment to enhance performance used stimuli that are perceptually unpleasant (e.g., click trains, binaural beats), the observation that music strongly affects neural oscillations^30,68,69^ motivates an approach whereby targeted acoustic modulation added to music might be used for neuromodulation, with particular goals outside the esthetic and/or social uses of music (i.e., ‘functional’ music rather than ‘art’ music). One barrier to this has been individual variability, as different neurotypes and cognitive functions may require a range of targets for their mechanisms. Here we targeted subpopulations (by ASRS/ADHD) with hypotheses based on theoretical considerations from systems neuroscience, and found effects on brain and behavior that have implications for the use of music to enhance cognition in everyday life. This suggests that amplitude modulations in music could be used specifically to mitigate the negative effects of cognitive failures in sustained attention that come from attentional difficulties.

## Methods

### Stimuli used in Experiments 1–3

For Experiments 1–3, we chose as background auditory stimuli two commercially-available tracks of music that were predicted to span different arousal levels while being similar in valence levels, in addition to a Pink noise control stimulus. Background auditory stimuli were selected from commercially available options used to help people focus while working. The tracks we used were: (1) ‘Techno March’ by Brain.fm (high-arousal track) and (2) ‘Tracking Aeroplanes’ by The Echelon Effect (low-arousal track). Our listener ratings (Fig. 2a) confirmed that the tracks differed in arousal but not in valence, suggesting that their effects on performance were attributable to arousal rather than valence. (3) Pink noise was also chosen as a control stimulus; it is often used for focused work while also being used in behavioral experiments because it has a spectrum that falls off with increasing frequency, similar to many auditory environments (unlike white noise which is spectrally flat). We limited our auditory stimuli to these three tracks in Experiments 1 in order to obtain experimentally well-validated auditory stimuli for neuroimaging and electrophysiology experiments in Experiments 2 and 3, both of which necessitated time-locked analyses on a smaller number of participants.

The tracks were musically dissimilar (to drive differences in valence and arousal) but in terms of low-level acoustic features they differed most strikingly in the modulation domain—the less arousing music was at a slow tempo and contained slow modulations, while the more arousing music was at a fast tempo and contains fast modulations. The difference in the tracks’ modulation characteristics can be visualized with the modulation spectrum (Fig. 1). The slow music contained a broad region of modulation energy from 0–8 Hz, while the fast music contained a clear peak around 14–16 Hz. This suggests tempo was not the sole cause of the difference in modulation characteristics, as tempo would simply shift the modulation spectrum. The difference in shape of the modulation spectrum instead reflects differences in the music, particularly note values (event durations). That is, the fast music was at a faster tempo, but also had faster note values. In particular, the modulation peak in the fast music was due to a consistent 32nd-note-rate amplitude modulation that had been deliberately applied as a feature of this kind of focus music. In contrast, the slow music contained long notes at several durations (e.g., whole, half) resulting in the absence of a single modulation peak. To complement the music tracks, the Pink Noise contained modulation energy broadly distributed in the higher modulation ranges (roughness). The modulation-domain differences were large and may partly underlie the music’s effects, but the tracks varied in other ways (tonality, instrumentation, etc.) which likely contributed to arousal. Due to the differences in frequency content of the three stimuli, rms-normalization was not appropriate (resulting in a large difference in perceived loudness across the stimuli); instead the stimuli were loudness-normalized by ear which produced rms values (average of left and right channels) of 0.059, 0.061, and 0.014 for AM+, Control − Music, and Pink Noise respectively.

### Experiment 1: Procedure

In Experiments 1 and 4 (the online behavioral experiments), users enrolled via Amazon’s Mechanical Turk platform, and provided informed consent as approved by New England IRB. All ethical regulations relevant to human research participants were followed. To enroll, users must be over 18 and have normal hearing by self-report. If they chose to participate in our experiment, they were directed to a cover page with consent documentation and a simple description of the task, followed by a page with payment information. If they still chose to participate, they initiated a volume calibration task in which they heard a train of 1 kHz tones at alternating levels, 10 dB apart. They were told to set the volume on their computer so that only every other tone was audible, and told not to change their volume after this calibration step. To ensure compliance a short task after the main experiment required the participant to count the audible tones in a decrementing series of 1 kHz tones (−5 dB per step); those who counted fewer tones than expected were excluded from analysis. Following the initial volume calibration participants were directed to a headphone screening task^70^ composed of six 3AFC questions (<1 min). If they passed the headphone screening they were directed to the main task instructions and could begin when ready.

In Experiment 1 our participants completed 1200 trials of a SART^26,27,71^. In this task, a single digit ranging from 0 to 9 appeared on the screen for each trial. Each digit was presented for 250 ms followed by a 900 ms mask, resulting in a 1150 ms inter-trial interval. Participants’ task was to respond to any digit except for 0. Instructions for the task were as follows: “Numbers will appear on the screen. If you see 1–9, hit any key; if you see 0 do not hit any key.” Participants were paid at a rate of $0.01 per correct response and -$0.10 per commission error (misses were given no pay; $0.00), resulting in an average of ~$12/h for the overall task. Participants are not penalized in any way for leaving, but any who did were not granted the performance bonus (this was communicated at the outset), and were excluded from analysis as incomplete data. Participants were told the experiment would run for about 20 min, and they should try to complete the entire experiment.

Valence and arousal ratings for these stimuli were obtained in a separate group of 62 participants under the same screening procedures. In this rating task, a webpage displayed several audio player bars, each corresponding to a music track. Under each player were two sliders (100-pixel resolution) with the ends of the sliders labeled ‘Positive’–‘Negative’ and ‘Calming’–‘Stimulating’. Participants were given as much time as they liked to listen to the tracks and decide on the placement of the sliders, and were permitted to move between tracks to better judge them relative to one another. The stimuli for Experiment 1 and Experiment 4 were rated for valence and arousal together (by the same participants).

### Experiment 1: Data analysis

Raw data from Amazon’s Mechanical Turk were exported to Matlab for analysis. The dependent variable was accuracy (d-prime), and independent variables were music condition and presentation order (6 blocks of 200 trials each; each music condition ran for 2 blocks). Listening volume varied somewhat across participants (See Supplementary Fig. 1 for this distribution) and was used as a covariate.

### Experiment 1: Participants

We used Amazon’s Mechanical Turk platform to recruit and enroll participants. One hundred sixty participants were recruited for Experiment 1 (82 male, 76 female, 2 other/chose not to respond; mean age = 45). 102 participants were recruited to obtain valence and arousal ratings for the stimuli used in Experiments 1 and 4 (obtained in the same participants). Participants were asked to wear headphones, and not to change their volume or turn off audio during the experiment. They were told that the background music was unrelated to the task, but that they should nonetheless ensure they could hear the background music, because it was needed to control the acoustic environment across participants. To ensure compliance we employed a headphone screening task^70^ as well as audio checks after each block, and a test of volume level at the end of the experiment. Participants who failed any of these were removed from data analysis. The final dataset for Experiment 1 comprised 83 of the initial 160 participants (52% passed screenings); the final dataset for the valence and arousal ratings comprised 62 of the initial 102 participants (60.7% passed screenings).

### Experiment 2: Procedure

In Experiments 2 and 3 (the neuroimaging experiments), participants provided informed consent as approved by the Northeastern University IRB. All ethical regulations relevant to human research participants were followed. The same three background music conditions from Experiment 1 (AM + Music, Control − Music, and Pink noise) were used in the fMRI study. During task fMRI, participants completed the SART while listening to AM + Music, Control − Music, and Pink noise in counterbalanced order. All experiment conditions were the same as Experiment 1 except inter-trial interval was 1425 ms, which was set to be equivalent to 3 TRs (as TR = 475 ms). Before each block the volume of the auditory stimulus was adjusted to a comfortable level (by communicating with the experimenter).

#### MRI acquisition

High-resolution T1 and functional images were acquired in a 3 T Siemens Skyra MRI scanner at the Olin Neuropsychiatry Research Center at the Institute of Living.

The anatomical images were acquired using a T1-weighted, 3D, magnetization-prepared, rapid-acquisition, gradient echo (MPRAGE) volume acquisition with a voxel resolution of 0.8 × 0.8 × 0.8 mm^3^ (TR = 2.4 s, TE = 2.09 ms, flip angle = 8°, FOV = 256 mm). Task functional MRI was acquired as 1268 contiguous fast-TR echo planar imaging functional volumes (TR = 475 ms; TE = 30 ms; flip angle = 90, 48 slices; FOV = 240 mm; acquisition voxel size = 3 × 3 × 3 mm^3^), resulting in a sequence that lasted approximately 10 min. Each background music condition was administered in a 10-min sequence, resulting in approximately 30 min of scan time for each participant.

### Experiment 2: Data analysis

#### MRI preprocessing

Task and structural MRI preprocessing was carried out using the SPM12 software (http://www.fil.ion.ucl.ac.uk/spm/) with the CONN Toolbox (http://www.nitrc.org/projects/conn)^72^. In order, this consisted of functional realignment and unwarp, functional centering, functional slice time correction, functional outlier detection using the artifact detection tool (http://www.nitrc.org/projects/artifact_detect), functional direct segmentation and normalization to MNI template, structural centering, structural segmentation and normalization to MNI template, and functional smoothing to an 8 mm gaussian kernel^73^. Denoising steps for functional connectivity analysis included correction for confounding effects of white matter and cerebrospinal fluid^74^, and bandpass filtering to 0.008–0.09 Hz.

#### Univariate task-fMRI analysis

Task fMRI analyses were done in SPM12^29^. Task fMRI analyses included: (1) Within-subject ANOVA comparing the three sessions (AM+, Control − Music, and Pink noise). (2) Hits, misses, false alarms, and correct rejections were separately modeled. (3) Seed-based functional connectivity was assessed using Conn and compared between the AM+, Control − Music, and Pink noise conditions. The resulting images were corrected at *p* < 0.05 FDR-corrected levels for both voxel-wise and cluster-wise comparisons.

### Experiment 2: Participants

34 Wesleyan undergraduates (16 males, 18 females; mean age = 20.4, SD = 1.94) participated for course credit.

### Experiment 3: Stimuli and procedure

Participants completed 680 trials of SART, a visual GO/NOGO task with inter-trial interval of 1150 ms, same as Experiment 1. This was done under four auditory conditions, presented in counterbalanced order: AM+, Silence (within subjects), Control − Music, and Pink noise (between subjects). EEG was recorded with a 64-channel BrainVision actiCHamp system with PyCorder software in a sound attenuated and electrically shielded chamber.

### Experiment 3: Data analysis

Behavioral data: RT coefficient of variation (SD/M) was calculated for every block of 10 trials.

EEG data were filtered using 0.5 Hz high pass filter and 60 Hz notch filter for electrical noise. Data were re-referenced to channels TP9 and TP10 and corrected for ocular artifacts using ICA. Matlab with EEGLAB toolbox^75^ were used for analyses.

Preprocessing: First, bad channels were rejected and then interpolated using EEGLAB’s rejchan and eeg_interp functions. Stimulus-brain coupling was assessed by first applying Morlet wavelet filtering at every single Hz from 1 to 50 Hz. Then, the Hilbert transform was done on each Hertz to get phase angle of stimulus and of EEG data, and the coupling between stimulus and EEG was assessed as the PLV where plv(ch) = abs(sum(exp(1i*(phase_filtered_EEG(ch,:) - phase_filtered_music)))). This was applied in parallel to EEG data and to sound stimuli. The Pink Noise stimulus was generated in real time using Max/MSP (i.e., was not a stored file) and so the stimulus-brain coupling analysis was not applied to the Pink Noise data.

### Experiment 3: Participants

Forty Wesleyan undergraduates (10 males; 30 females; mean age = 19, SD = 0.75) participated for course credit.

### Experiment 4: Stimuli

The stimuli (background music) were based on two different musical tracks; each had variants created that added amplitude modulation at three rates (8, 16, 32 Hz) and depths (low, medium, high). Modulation depth differences were quantified after processing to account for interactions between the music and modulator. We used the difference between original and processed tracks’ modulation spectra (in each cochlear channel; Fig. 7) as a metric of applied modulation depth, and set the modulator such that our three depth conditions stepped up evenly in terms of this metric going from low to high depth. The transitions between conditions were implemented with smooth crossfades preserving track location (rather than a break and starting the music from the beginning).

Modulation patterns were aligned to the metrical grid of the music. This scheme meant that the relationship of the underlying music to the added modulation was as consistent as possible over very different rates. This is desirable in controlling for differences between conditions that arise from intrinsic properties of the underlying (pre-modulation) acoustic signal. For example, a musical event (e.g., a drum hit) transiently amplified by a modulation peak in the 8 Hz condition would also be so in the higher-rate conditions. This is only the case because the different modulation rates are aligned to the music and are integer multiples of each other.

As can be seen in Fig. 7, the applied modulation differences exist predominantly in the low-mid range of the frequency spectrum, with little modulation difference at high frequencies. This was by design, due to esthetic considerations given the spectrotemporal density of the underlying music: Modulation applied in a broadband manner tended to interact with sparse events in the high frequency regions, which was occasionally annoying (salient). We confined added modulation to lower frequencies by applying it to only the frequency range 200 Hz to 1 kHz.

Acoustic analysis before and after processing showed that the manipulated stimuli differed from the originals only in the modulation domain, and not in the audio frequency spectrum. Our conditions were therefore identical in terms of musical content and spectral balance (‘EQ’), eliminating important confounding factors and ensuring any behavioral differences can be attributed to applied modulation alone.Due to the similarity across the stimuli they were simply peak-normalized to 0.5, giving rms levels (average across left and right channels) in all cases between 0.080 and 0.092, where the faster and higher-depth modulation conditions had lower rms levels and the unmodulated tracks had the highest rms levels.

### Experiment 4: Procedure

Experimental procedure in Experiment 4 was similar to Experiment 1 with the addition of a fourth block of trials. The total number of trials in Experiment 4 was the same as in Experiment 1 (1080 trials) and so each block in Experiment 4 was 270 trials (~5 min).

### Experiment 4: Data analysis

For Experiment 4, a mixed-effects ANOVA was run with the dependent variable of d-prime difference relative to the no-modulation control condition. Independent variables were the within-subjects factor of modulation (3 levels for each within-subjects manipulation: 8 Hz, 16 Hz, and 32 Hz for the modulation-rate manipulation; low, medium, and high depth for the modulation-depth manipulation) and trial block (early and late blocks respectively). ASRS score was treated as a between-subjects covariate.

### Experiment 4: Participants

Recruitment and experimental procedure (including screening) was identical to Experiment 1. Experiment 4 involved 221 participants (120 males; 99 females; 2 other/chose not to respond; mean age = 36, SD = 11.10). The final dataset for Experiment 4 comprised 175 of the initial 221 participants (79% passed screenings).

### Statistics and reproducibility

The valence-arousal data for the music stimuli (*N* = 62) were analyzed with a multivariate one-factor MANOVA, with music type as the within-subjects factor. Behavioral data (Experiments 1 and 4; *N* = 83 and *N* = 175 respectively) were analyzed with a repeated measures mixed-effects ANOVA with the dependent variable of d-prime. fMRI data (Experiment 2; *N* = 34) were analyzed with a within-subjects ANOVA (with cluster-wise FDR correction) comparing overall brain activity during the three conditions. To relate behavior to brain activity during the different music tracks, we fit separate parametric models in SPM12^29^ for hit trials and false alarm trials for each auditory condition. EEG data (Experiment 3; *N* = 40) were analyzed by examining the coupling between the EEG and the acoustic signal, computing the stimulus-brain PLV for every frequency (in 1-Hz bins). Change in phase-locking over time was analyzed using *t*-tests comparing late trials vs. early trials in each channel (FDR-corrected).

## Supplementary information

Supplementary Information

Description of Additional Supplementary Files

Supplementary Data 1

## Data Availability

The neuroimaging data (EEG and fMRI) which are very large are available from the corresponding author upon reasonable request. Behavioral data are available in the following OSF repository: 10.17605/OSF.IO/4V8AN. Numerical source data for all graphs in the manuscript can be found in Supplementary Data 1 file.

## Code availability

Behavioral data was processed with Matlab 2021. EEG data was processed with Matlab and EEGLAB toolbox. fMRI data was processed with SPM12. Code used in analysis is available from the corresponding author upon reasonable request.
