# Author Correction: Rapid modulation in music supports attention in listeners with attentional difficulties

**DOI:** 10.1038/s42003-025-08777-3

**Published:** 2025-09-08

**Authors:** Kevin J. P. Woods, Gonçalo Sampaio, Tedra James, Emily Przysinda, Bernardo Cordovez, Adam Hewett, Andrea E. Spencer, Benjamin Morillon, Psyche Loui

**Affiliations:** 1 Brain.fm, Brooklyn, NY USA; 2 Wesleyan University, Middletown, CT USA; 3 RootBio LLC, Pacifica, CA USA; 4 Boston University Chobanian & Avedisian School of Medicine, Boston, MA USA; 5 Aix Marseille Université Inserm, INS, Institut de Neurosciences des Systèmes, Marseille, France; 6 Department of Music, College of Arts, Media, and Design, Northeastern University, Boston, MA USA

**Keywords:** Attention, Cognitive control, Human behaviour

Correction to: *Communications Biology* 10.1038/s42003-024-07026-3, published online 23 October 2024

In the version of the article initially published, Bernardo Cordovez was not included in the author list, as is now amended in the HTML and PDF versions of the article.

