# Author Correction: Rapid modulation in music supports attention in listeners with attentional difficulties

**DOI:** 10.1038/s42003-026-10015-3

**Published:** 2026-04-13

**Authors:** Kevin J. P. Woods, Gonçalo Sampaio, Tedra James, Emily Przysinda, Bernardo Cordovez, Adam Hewett, Andrea E. Spencer, Benjamin Morillon, Psyche Loui

**Affiliations:** 1 Brain.fm, Brooklyn, NY USA; 2 Wesleyan University, Middletown, CT USA; 3 RootBio LLC, Pacifica, CA USA; 4 Boston University Chobanian & Avedisian School of Medicine, Boston, MA USA; 5 Aix Marseille Université Inserm, INS, Institut de Neurosciences des Systèmes, Marseille, France; 6 Department of Music, College of Arts, Media, and Design, Northeastern University, Boston, MA USA

**Keywords:** Attention, Cognitive control, Human behaviour

Correction to: *Communications Biology* 10.1038/s42003-024-07026-3, published online 23 October 2024

In the version of the article initially published, the “Competing interests” section read “The authors declare no competing interests” but should have read “K.J.P.W. is an employee of Brain.fm, Inc., the company that produced the music used in the modulated-music conditions of this study. K.J.P.W. also holds unexercised equity options in Brain.fm, Inc. A.H. was an employee of Brain.fm, Inc. and held equity in the company at the time this research was conducted. The remaining authors declare no competing interests.” This correction has been made to the HTML and PDF versions of the article.

