# Supplementary material for: Rapid modulation in music supports attention in listeners with attentional difficulties
Source: Commun Biol. 2024 Oct 23;7:1376. doi: 10.1038/s42003-024-07026-3 (PMC11499863; doi:10.1038/s42003-024-07026-3)
Supplement: Supplementary file 1 — Supplementary Information [file 42003_2024_7026_MOESM1_ESM.pdf]

Supplementary Items

Listener volume settings. Online participants were asked to first calibrate their volume with an alternating sequence of low-level tones separated by 10dB; they were told to set their computer volume so that only half the beeps could be heard. This approximate calibration was intended to somewhat narrow the distribution of volume settings participants used. In a post-test for volume, participants counted the audible tones in a series decrementing by 5dB per tone.

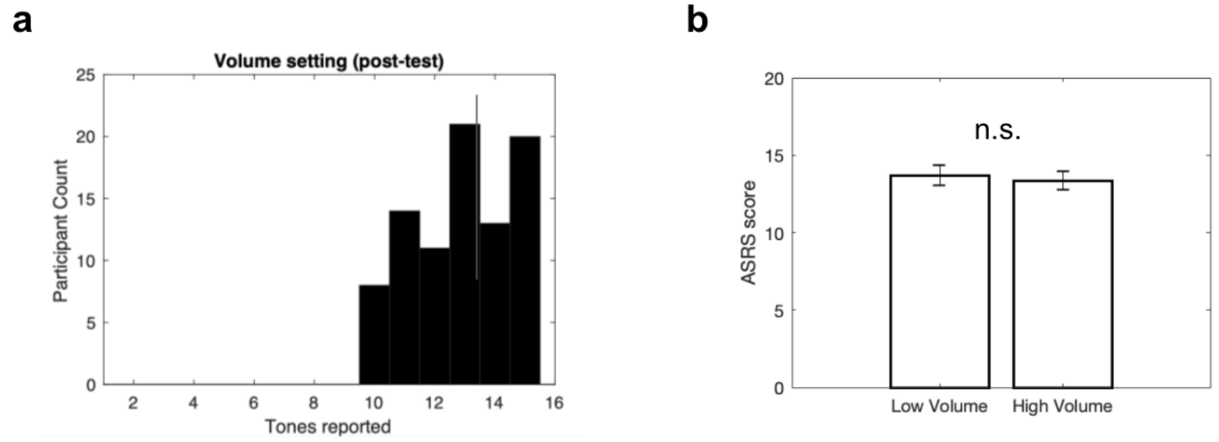

Supplementary Figure 1. Online volume settings. a, Relative volume distribution for participants in Experiment 1 (N=83); measured as the distribution of tones counted in a volume-setting post-test where 15 tones decremented in volume by 5db per tone. **b,** ASRS scores across participants in the high- and low-volume groups.

Volume level might be expected to influence levels of arousal, or the effect of music in general [76]. To see if volume levels interact with the factors addressed in our main results, we split our participants into two groups based on their volume settings (closest median split) and noted that the ASRS scores did not differ between the high-volume and low-volume listeners (Fig S1B).

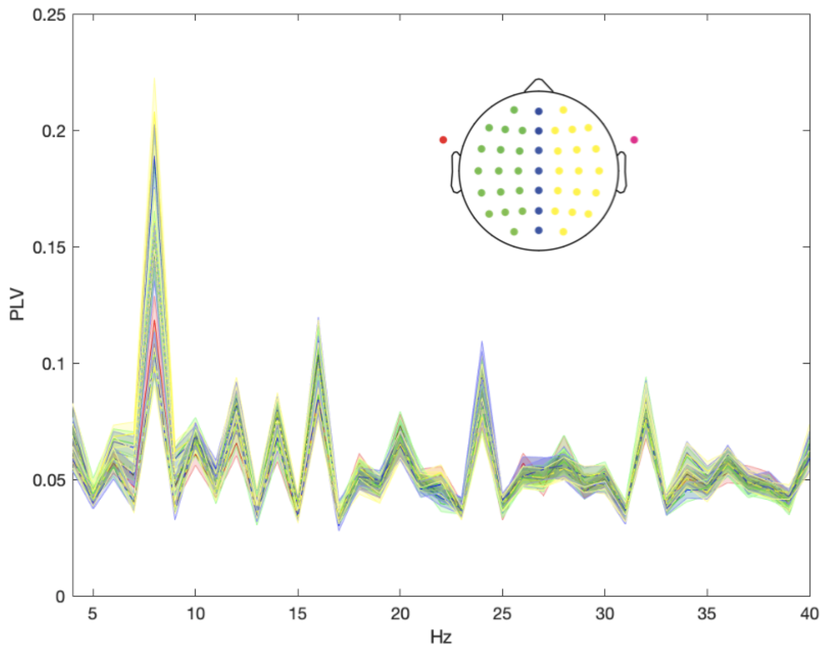

Supplementary Figure 2. Stimulus-brain coupling across EEG electrode channels. For Experiment 3, PLV from EEG data are separately plotted here for left (green), midline (blue), and right (yellow) channels for frontal lateral sites (solid lines), frontal sites (dotted lines), and parietal sites (dashed lines). Results show largely similar stimulus-brain coupling across multiple recording sites.
